# Using a Mathematical Model to Analyze the Role of Probiotics and Inflammation in Necrotizing Enterocolitis

**DOI:** 10.1371/journal.pone.0010066

**Published:** 2010-04-19

**Authors:** Julia C. Arciero, G. Bard Ermentrout, Jeffrey S. Upperman, Yoram Vodovotz, Jonathan E. Rubin

**Affiliations:** 1 Department of Mathematics, University of Pittsburgh, Pittsburgh, Pennsylvania, United States of America; 2 Department of Surgery, University of Southern California, Los Angeles, California, United States of America; 3 Department of Surgery, University of Pittsburgh, Pittsburgh, Pennsylvania, United States of America; University of Giessen Lung Center, Germany

## Abstract

**Background:**

Necrotizing enterocolitis (NEC) is a severe disease of the gastrointestinal tract of pre-term babies and is thought to be related to the physiological immaturity of the intestine and altered levels of normal flora in the gut. Understanding the factors that contribute to the pathology of NEC may lead to the development of treatment strategies aimed at re-establishing the integrity of the epithelial wall and preventing the propagation of inflammation in NEC. Several studies have shown a reduced incidence and severity of NEC in neonates treated with probiotics (beneficial bacteria species).

**Methodology/Principal Findings:**

The objective of this study is to use a mathematical model to predict the conditions under which probiotics may be successful in promoting the health of infants suffering from NEC. An ordinary differential equation model is developed that tracks the populations of pathogenic and probiotic bacteria in the intestinal lumen and in the blood/tissue region. The permeability of the intestinal epithelial layer is treated as a variable, and the role of the inflammatory response is included. The model predicts that in the presence of probiotics health is restored in many cases that would have been otherwise pathogenic. The timing of probiotic administration is also shown to determine whether or not health is restored. Finally, the model predicts that probiotics may be harmful to the NEC patient under very specific conditions, perhaps explaining the detrimental effects of probiotics observed in some clinical studies.

**Conclusions/Significance:**

The reduced, experimentally motivated mathematical model that we have developed suggests how a certain general set of characteristics of probiotics can lead to beneficial or detrimental outcomes for infants suffering from NEC, depending on the influences of probiotics on defined features of the inflammatory response.

## Introduction

Necrotizing enterocolitis (NEC) is a severe disease of the gastrointestinal (GI) tract that is characterized by increased permeability of the intestine and is primarily observed in pre-term babies. Although the causes of this disease are not fully known, most studies conclude that prematurity is the greatest risk factor. NEC affects 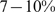 of low birth weight (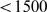 g) premature infants and is observed typically within 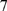 to 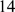 days of birth [1]. Symptoms of NEC mainly involve gastrointestinal dysfunction, such as abdominal distension and feeding intolerance. Current forms of treatment may be invasive, including surgical interventions, and are often insufficient due to the fragility of the patients and rapid progression of the disease. Mortality from NEC is nearly 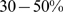 for infants with surgical intervention [2]. Moreover, infants who recover from severe forms of the disease may experience complications and other bowel disorders later in life [3]–[8]. The severity of this disease, which stems from a complex inflammatory response and immaturity of organ architecture and physiology, coupled to a lack of effective therapy, suggests that systems approaches such as computational modeling may be necessary to gain a fuller insight into both mechanism and therapy.

### Possible factors contributing to NEC

Although its pathophysiology is not entirely understood, NEC is thought to be related to the physiological immaturity of the GI tract and altered levels of normal flora in the intestines. A mature intestine contains many defense mechanisms that act as barriers to harmful bacteria. Many of these defense mechanisms, such as peristalsis and tight junctions between intestinal epithelial cells [1], [3], [4], are abnormal or decreased in an immature intestine, and thus bacteria normally confined to the intestinal lumen are able to reach systemic organs and tissues. Bacterial translocation triggers the activation of the inflammatory response, which leads to further epithelial damage [3], [9]. The inflammatory response is often exaggerated in premature infants due to a lack of differentiation between harmful and beneficial bacteria [1], [3].

An abnormal pattern of bacterial colonization in pre-term infants may also contribute to the pathogenesis of NEC. Colonization by normal (ostensibly beneficial) flora such as *Bifidobacterium* and *Lactobacillus* is necessary for the normal development and protective function of the newborn intestine [1], [4], [10], [11]. Premature infants in the neonatal intensive care unit are more likely than other infants to be colonized by pathogenic bacteria due to the use of antibiotics and feeding instrumentation. In addition, formula-fed infants are colonized with a complex flora containing a much lower amount of *Bifidobacteria* than the amount found in breast-fed infants, and indeed, pre-term infants fed formula have significantly higher rates of NEC than those fed breast milk [12].

Recently, Toll-like receptor-4 (TLR-4) has been shown to be significantly increased in mice and humans with NEC compared with healthy infants [13]. Since TLR-4 expression can cause increased apoptosis of intestinal epithelial cells and reduced intestinal healing, TLR-4 signaling may also play a significant role in the development of NEC. Together, immaturity of the GI tract and the inflammatory response, abnormal intestinal bacterial colonization, and altered TLR-4 signaling at least partly account for the increased risk for pre-term babies to develop NEC.

### Possible treatment for NEC

Given this growing understanding and identification of the factors that contribute to NEC, it seems important to develop treatment strategies aimed at bolstering the integrity of the epithelial wall, preventing excessive inflammation, and limiting the presence of pathogenic bacteria. One proposed treatment method is the administration of probiotics, which are defined as non-pathogenic species of bacteria that promote the health of the host [14]. Probiotics used to treat NEC consist mainly of *Bifidobacterium* and *Lactobacillus.* Probiotics compete with pathogenic bacteria for host binding sites and nutrients while also stimulating host defense mechanisms and enhancing intestinal maturation. Probiotic bacteria can protect against systemic bacterial invasion by decreasing the permeability of the gastrointestinal wall [10], [11].

Several studies have shown a reduced incidence and severity of NEC in neonates treated with probiotics [12], [14]–[20]. Hoyos et al. [18] noted an almost threefold reduction in the incidence of NEC after the administration of probiotics that included *Lactobacillus acidophilus* and *Bifidobacterium infantis*. Infants treated with a probiotic mixture in two separate studies [19], [20] showed a reduced incidence of NEC and decreased disease severity. Despite these trends, the appropriate timing and dosing of probiotic administration have not been determined. In addition, questions regarding the safety and efficacy of delivering probiotic bacteria to pre-term infants have not been fully answered, since not all studies have shown beneficial effects of probiotics. In a study by Dani et al. [21], infants treated with *Lactobacillus* were shown to have an increased incidence of sepsis, and the observed decrease in NEC incidence was not statistically significant. Similarly, Land et al. [22] observed cases of *Lactobacillus* sepsis in infants treated with probiotics. However, lactobacillemia can occur naturally and thus may or may not have been related to probiotic treatment.

### Current model

Experimental studies have shown a potential clinical benefit of probiotics in NEC patients but have not identified the mechanisms underlying the efficacy of probiotic treatment. It is hypothesized that probiotics improve the barrier function of the intestine by increasing transepithelial resistance, protecting against cell death, inducing specific mucus genes, and stimulating the production of nonfunctional receptor decoys in the intestinal lining [1], [4]. Probiotics have also been shown to decrease cytokine activation so as to prevent an exaggerated inflammatory response [1] and to inhibit TLR-4 expression so as to reduce the development of NEC [13].

We hypothesized that the protective potential of these mechanisms can be analyzed using a mathematical model. Building upon insights established by theoretical models of the acute inflammatory response [9], [23]–[26], the current study aims to analyze the impact of pathologic bacteria in the context of NEC, as motivated by Hunter et al. [27], and to predict the conditions under which probiotics may be successful in promoting the health and survival of infants at risk for NEC. Pathogenic and probiotic bacteria populations initially present in the intestinal lumen are simulated using an ordinary differential equation model. The degree of intestinal wall permeability is a variable in the system that corresponds indirectly to the role that Damage-associated Molecular Pattern (DAMP) molecules play in propagating the positive feedback between inflammation and damage [28], [29]. Based on this permeability, the conditions leading to bacterial translocation into the systemic circulation can be predicted. In the model, the inflammatory response targets pathogens while simultaneously causing increased damage to the intestinal wall. System behavior in the presence and absence of probiotics is compared, and the relative therapeutic contributions of various hypothesized effects of probiotics are analyzed. Since predicted health and disease states are shown to be sensitive to the initial degree of infection and virulence of the pathogen, the model can be used to define a set of conditions under which clinical studies should be conducted to identify the situations in which probiotic treatment is beneficial and to optimize probiotic administration.

## Methods

**Figure 1:**
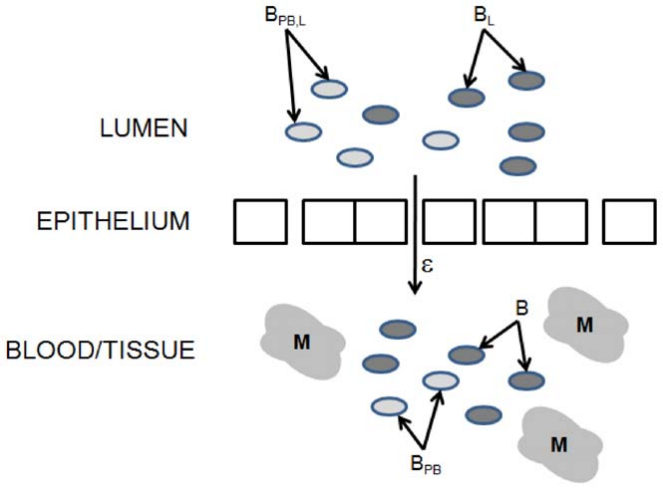
Schematic diagram of compartmental model for necrotizing enterocolitis. Two compartments are considered: the intestinal lumen and a combined blood/tissue compartment. 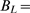
pathogenic bacteria in the lumen. 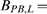
probiotic bacteria in the lumen. 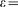
permeability of epithelial wall. 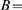
pathogenic bacteria in the blood/tissue. 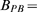
probiotic bacteria in the blood/tissue. 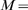
immune cells in the blood/tissue.

A system of ordinary differential equations is used to track both pathogenic and probiotic bacteria in two compartments: an intestinal lumen compartment and a combined blood/tissue compartment (see Figure 1). The rate of “leakiness,” or permeability to bacteria (i.e., efflux of bacteria), of the intestinal epithelial layer is treated as a variable. Initially, both pathogenic and probiotic bacteria are present only in the lumen. Transport of these populations into the blood/tissue compartment is assumed to occur across weakened tight junctions in the epithelium due to the immaturity of the gut [3], through damaged regions of the epithelium (induced by inflammation), or via Toll-like receptors (e.g., TLR-4) [4]. Immune cells are present in the blood/tissue region and become activated once bacteria enter the blood/tissue region. The success of the inflammatory response in eliminating pathogens comes at the cost of additional damage that the inflammatory response causes to the intestinal wall. Bacterial permeability is assumed to increase in proportion to the inflammatory response.

The majority of the parameter values for this model are taken directly from two previous models of the inflammatory response [9], [23]. The remaining unknown parameters are approximated according to experimental observations and biological assumptions. Table 1 gives a list of the different populations that are tracked by this model, and Table 2 gives the values, descriptions, and sources of the model parameters.

**Table 1:** Variables for NEC model.

Variable	Description
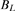	Pathogenic bacteria in the intestinal lumen
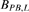	Probiotic bacteria in the intestinal lumen
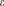	Permeability of intestinal wall to bacteria
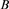	Pathogenic bacteria in the blood/tissue
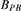	Probiotic bacteria in the blood/tissue
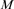	Activated inflammatory cells

**Table 2:** Parameter values for NEC model.

Parameter	Value	Unit	Description	Source
r 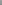	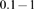	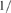 h	growth rate of pathogenic bacteria in lumen	[9]
r 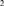	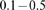	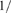 h	growth rate of probiotic bacteria in lumen	
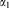	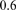		competitive effect of B 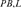 on B 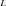 in lumen	
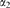	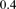		competitive effect of B 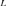 on B 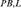 in lumen	
K 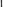	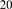	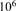 cells 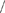 g	carrying capacity of B 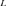	[9]
K 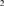	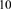	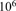 cells 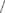 g	carrying capacity of B 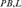	
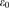	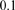	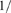 h	baseline rate of bacterial translocation	[30]
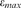	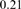	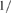 h	maximum rate of bacterial translocation	[30]
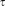	24	h	time scale for epithelium repair	
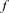	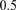	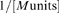	effect of inflammatory response on permeability	
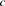	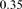	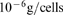	effect of probiotics on permeability	
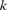	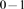		contribution of probiotics to threshold crossing	
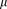	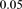	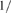 h	decay rate of inflammatory cells	[23]
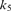	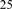	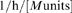	rate of destruction of pathogen by M	
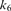	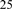	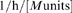	rate of destruction of probiotic bacteria by M	
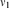	0.08	[M units]/h	source of inflammatory cells	[9]
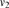	0.12	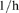	decay of inflammatory cells	[9]
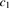	0.1	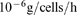	rate of inflammatory cell activation due to pathogen	[9]
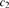	0.01	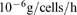	rate of inflammatory cell activation due to probiotics	[9]

### Intestinal lumen compartment

In the intestinal lumen, pathogenic bacteria (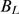) and probiotic bacteria (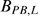) are assumed to compete with each other for resources and nutrients. This process is modeled using a competitive logistic interaction in equations (1) and (2).
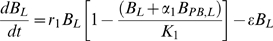
(1)

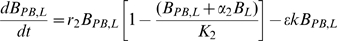
(2)

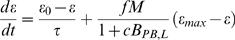
(3)The pathogenic and probiotic bacteria populations have growth rates 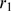 and 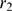 and carrying capacities 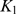 and 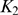, respectively. In this model, the carrying capacity of pathogenic bacteria is assumed to be higher than that of probiotic bacteria, 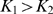. Probiotic bacteria are assumed to have a strong effect on the growth rate of pathogenic bacteria, and thus the competition parameters 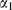 and 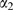 in equations (1) and (2) satisfy 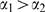. The second term in each of equations (1) and (2) describes the transfer of bacteria populations from the lumen into the blood/tissue compartment, which depends on the intestinal wall permeability. The rate of bacterial efflux through the intestinal wall is given by 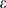 and is tracked in equation (3). The model is used to study scenarios of health and disease in premature infants. Bacterial permeability is initially given by a low but nonzero value, 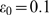 h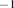. Physiologically, this baseline permeability would correspond to a gut lining that is not fully developed or to an initial breakdown in the intestinal barrier due to the activation of TLR-4. Even in mature infants, baseline intestinal permeability would not be zero since the model should accomodate the possibility that a sufficiently large bacterial insult will lead to bacterial translocation and blood infection. Also, animal studies have suggested that the intestinal lining is permeable to fluorescein isothiocyanate-labeled dextran even under control conditions [30]. Despite the nonzero initial condition for bacterial permeability, if the levels of pathogenic and probiotic bacteria in the lumen remain sufficiently low, bacteria are assumed not to translocate into the blood and tissues. The parameter 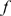 in equation (3) indicates the extent to which epithelial damage is caused by the inflammatory response. Since probiotics have been shown to enhance the viability of the intestinal barrier [1], [10], [11], parameter 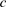 is varied in the system to assess the potential beneficial effect of probiotics on intestinal wall permeability. Parameter 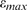 is defined as the maximum possible rate of bacterial permeability and has value 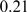 h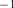. In a study by Han et al. [30], ileal permeability in mice increased slightly more than two-fold in the presence of lipopolysaccharides (LPS) with time; this provides an experimental basis for the ratio of 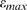 to 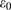 used in our model.

### Blood/tissue compartment

Equations (4)–(6) represent the evolution of pathogenic bacteria (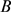) and probiotic bacteria (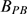) in a lumped blood/tissue compartment.
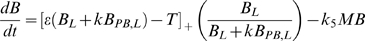
(4)

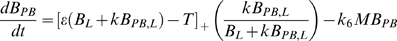
(5)

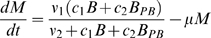
(6)


We assume that the rate at which bacteria enter this combined compartment depends on the permeability of the epithelial layer as well as on the number of bacteria present, relative to a threshold 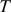. The threshold corresponds biologically to the resistance provided by the intestinal wall to the translocation of bacteria and is motivated by an experiment [30] in which the number of bacteria that permeated the intestinal wall was shown to increase as a step-function with time: after 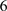 hours, no bacteria had entered the systemic circulation, but after 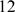 hours, the number of bacteria that permeated the intestinal wall increased sharply and remained at this maximum value for an additional 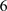 hours. This experimental observation is captured using the function 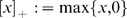. The threshold term 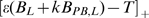 in each of equations (4) and (5) is multiplied by a ratio to ensure that the only source of pathogenic (probiotic) bacteria entering the blood/tissue compartment is the pathogenic (probiotic) bacteria in the lumen.

Biologically, it is unclear if pathogenic bacteria and probiotic bacteria are equally effective at breaching the epithelial barrier. Since probiotics are typically considered as beneficial to the host, it is hypothesized that more probiotic bacteria than pathogenic bacteria must be present in the lumen in order to exceed the threshold and enter the blood/tissue. In support of this hypothesis, Hooper and Macpherson [31] suggest that, under most circumstances, the number of commensal bacteria never reaches the threshold of triggering a systemic immune response. To explore this concept in the current model, a parameter 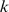 that varies between 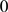 and 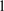 is used to scale the contribution of probiotic bacteria to exceeding threshold and triggering bacterial translocation from the lumen into the blood/tissue compartment. If 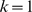, then pathogenic and probiotic bacteria are equally able to enter the blood/tissue, whereas if 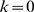, then only pathogenic bacteria will breach the epithelial layer. Although a single species of bacteria has not been associated with all cases of NEC, the Gram-negative *Enterobacteriaceae* are the most common species isolated from infants with NEC [4]. Normal flora such as *Bifidobacterium* and *Lactobacillus* are Gram-positive. This difference in bacterial type may partially explain the difference in the body's reactions to harmful and beneficial bacteria. Moreover, as described by Hooper and Macpherson [31], a particular bacterial species can range significantly between benign and pathogenic, promoting health in some cases but causing harm in others.

Pathogenic and probiotic bacteria are assumed to be destroyed by activated inflammatory cells (M) in the blood/tissue at rates 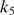 and 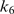, respectively. In equation (6), inflammatory cells are assumed to be activated by both pathogenic bacteria and probiotic bacteria. We hypothesize that pathogenic bacteria exert a stronger influence than probiotic bacteria on inflammatory cell activation [32], represented by 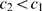. Finally, inflammatory cells are assumed to decay/die with rate 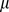.

## Results

To investigate various features of probiotic treatment for NEC, we first consider equations (1)–(6) in the absence of probiotics for varying levels of initial pathogenic insult, 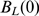. Next, the effects of probiotics on the growth of pathogenic bacteria in the lumen, on the permeability of the epithelial wall, and on the activation of the inflammatory response are analyzed. The mechanisms underlying the beneficial effects of probiotic treatment are investigated, and the components of an ideal probiotic treatment strategy are summarized.

Inspection of system (1)–(6) shows that model steady states can take two forms, one with baseline bacterial permeability (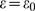) and no bacteria present in the blood/tissue compartment, and another with an elevated bacterial permeability and a nonzero presence of bacteria in the blood/tissue compartment. We refer to the former as the health state and the latter as the disease state.

### Model predictions in the absence of probiotics

**Figure 2:**
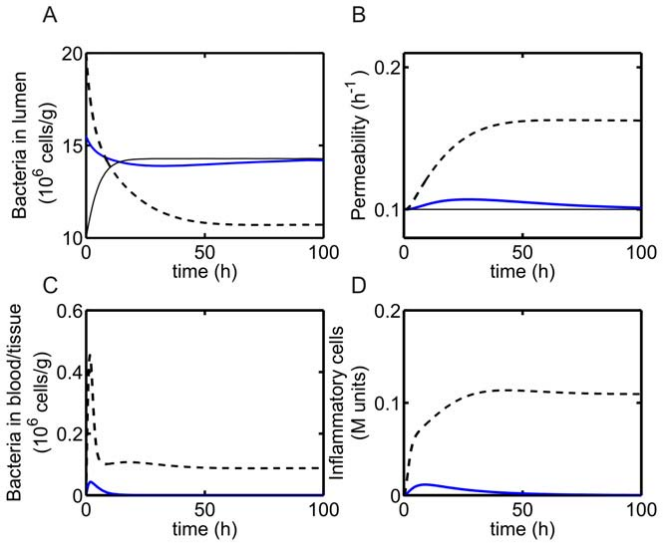
System dynamics in the absence of probiotics. Health or disease states are predicted as the initial level of pathogenic bacteria is varied: 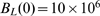 cells/g (thin curve, health), 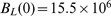 cells/g (thick blue curve, health), and 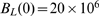 cells/g (dashed curve, disease). The growth rate of pathogenic bacteria is 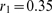 h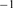 and the threshold is 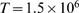 cells/g/h. (A) Bacteria in lumen. (B) Permeability. (C) Bacteria in blood/tissue. (D) Inflammatory cells.

In the absence of probiotics in the system, 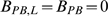. The thin curves in Figure 2 illustrate that a health state is maintained if a low level of pathogenic bacteria, 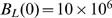 cells/g, is initially introduced with a pathogenic bacteria growth rate (virulence) of 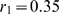 h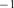 and a threshold of 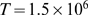 cells/g/h. Since the product of the bacterial permeability rate and the level of pathogenic bacteria in the lumen 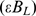 does not exceed 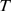, the levels of bacteria and inflammatory cells in the blood/tissue are zero (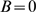 and 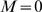) for all time, and the bacterial permeability remains at its baseline value. If the initial level of bacteria in the lumen is increased, for example to 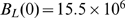 cells/g, then 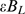 is initially above threshold and bacteria enter the blood/tissue; however, the infection is successfully cleared in the blood/tissue region by the inflammatory cells and a health steady state is restored (Figure 2, thick, blue curve). If a sufficiently large number of pathogenic bacteria is initially present in the system (e.g., 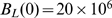 cells/g), then the threshold value is exceeded. A disease state is predicted, since pathogenic bacteria are never entirely cleared from the blood/tissue compartment and inflammation persists (Figure 2, dashed curve). Thus, we observe bistability of steady states in the system for 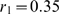 h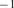.

**Figure 3:**
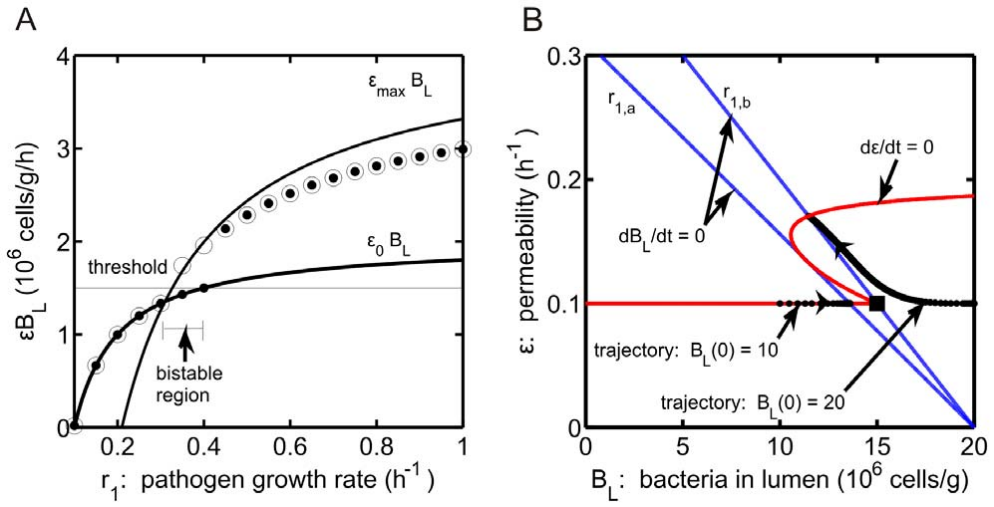
Steady state predictions in the absence of probiotics. (A) Steady state values of pathogenic bacteria and permeability as the growth rate of pathogenic bacteria (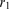) is varied. Steady state solutions of 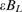 are given by (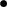) for 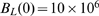 cells/g and () for 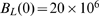 cells/g. In the bistable region, steady state solutions are exactly 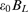 or close to 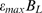 (curves labeled) depending on the initial level of pathogenic bacteria. Thin horizontal line: threshold, 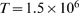 cells/g/h. (B) 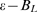 phase plane corresponding to system dynamics in panel A. A region of bistability is predicted when the 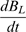 (blue) and 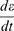 (red) nullclines intersect three times. This occurs for values of 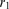 within 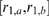 (corresponding nullclines included). Trajectories for 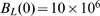 cells/g when 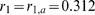 h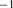 and 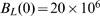 cells/g when 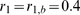 h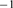 are also shown. The closed square gives the value of bacteria at which threshold is exceeded and bacteria are able to translocate into the blood/tissue.

In Figure 3A, the steady state values of 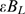 are plotted as a function of the pathogenic growth rate, 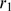, for two different initial conditions: 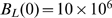 cells/g (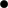) and 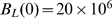 cells/g (). The solid curves correspond to 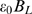 and 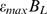, which are the theoretical lower and upper bounds on 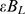 in steady state, and the thin horizontal line is the value of the threshold parameter 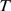. For consistency, a health steady state with 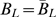 can only exist if 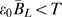, while a disease steady state with 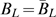 and 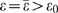 can only exist if 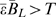. For both initial conditions, simulations yield convergence to a health state if 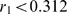 h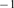 and convergence to a disease state for 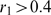 h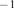. Interestingly, both health and disease steady states are stable for 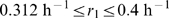. For values of 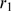 in this range, a disease state is predicted if 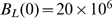 cells/g whereas a health state is predicted if 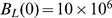 cells/g.

The bistable region can be identified precisely using the 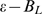 phase plane shown in Figure 3B. The slope of the 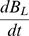 nullcline depends on 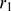 and determines the intersection point of the 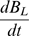 (blue) and 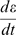 (red) nullclines. We define 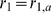 to be the infimum of the set of 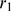 values at which the nullclines intersect three times and 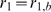 to be the supremum of this set. For values of 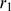 outside of 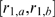, the nullclines intersect only once: for 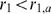 a health state is always predicted, and for 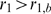 a disease state is always predicted. For 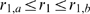, selection of health or disease depends on the initial bacterial insult, 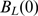. The nullclines corresponding to 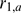 and 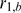 are labeled in Figure 3B, and sample trajectories (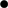) using the initial conditions from Figure 3A are also shown. The square on the 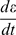 nullcline represents the point at which bacteria exceed threshold and translocate into the blood/tissue compartment (i.e., 
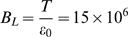
 cells/g). At this point, the equation defining the 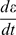 nullcline changes from 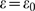 (below threshold) to 
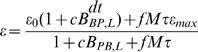
 (above threshold).

In summary, Figure 3 illustrates the mechanisms underlying the steady state outcomes in the model in the absence of probiotics and the dependence of the model prediction of health or disease on the initial pathogen level and pathogen growth rate 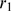. In particular, the bistability evident in Figure 3 arises in a parameter regime in which the inherent growth rate of the pathogenic bacteria population does not allow those bacteria to exceed the threshold level required to enter the blood/tissue compartment. Yet, if a sufficient number of pathogenic bacteria is introduced from an outside source, a sustained blood/tissue infection will result. We shall see that this bistability persists when probiotics are included in the model and plays a crucial role in how probiotics affect steady state outcomes.

### Model predictions in the presence of probiotics

**Figure 4:**
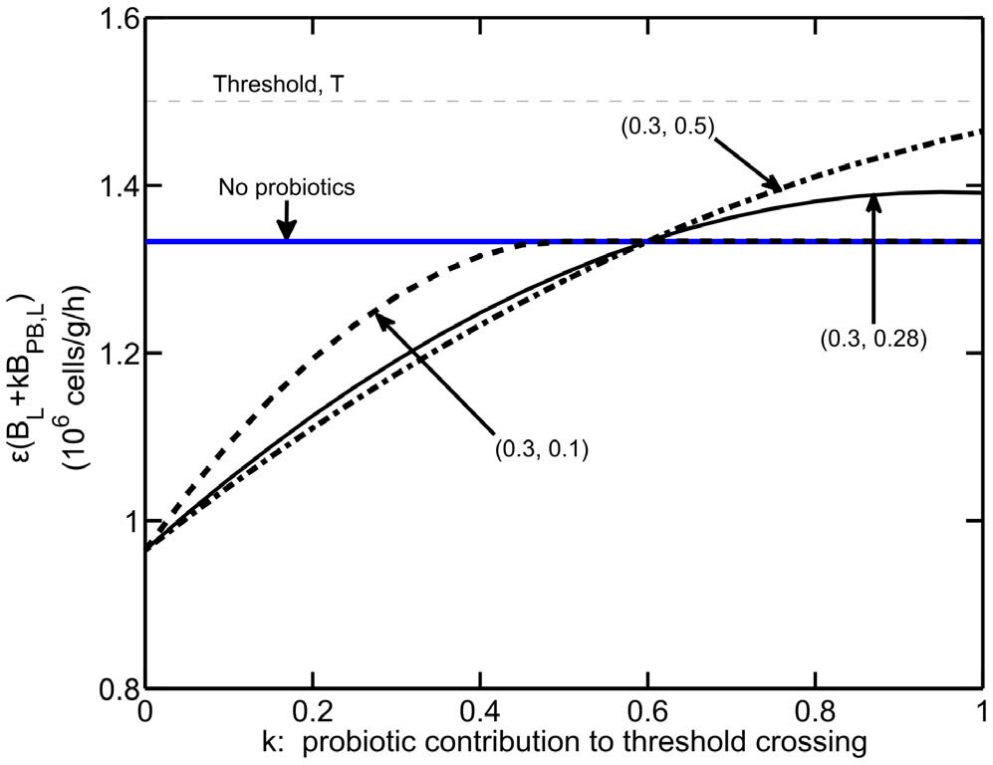
Steady state values of 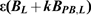 in the absence and presence of probiotics for varied 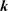 values. Thick, blue line: steady state value of 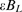 (no probiotics, labeled). Thin, dashed line: threshold value, T. Steady state values of 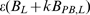 are shown for a small initial bacterial insult (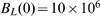 cells/g) and the following parameter combinations: 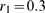 h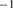 and 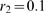 h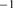 (dashed curve), 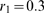 h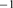 and 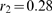 h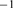 (solid curve), and 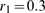 h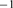 and 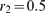 h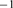 (dashed-dotted curve). Note, parameters are labeled as (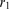,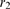) on the figure.

The addition of probiotics as a treatment method has two important effects on the system: probiotics compete with pathogenic bacteria in the lumen and they reduce the permeability of the intestinal wall. These effects are captured in the model by parameters 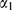 and 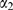 in equations (1) and (2) and by parameter 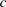 in equation (3). Parameter 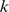 also encodes an important aspect of the hypothesized effect of probiotics in the system. Bacteria enters the blood/tissue compartment if 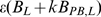 exceeds the threshold; 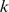 provides a measure of the contribution of probiotics to crossing the threshold. In Figure 4, the steady state values of 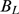, 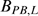, and 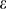 are calculated, and 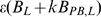 is plotted as a function of 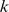 in the presence (dashed, solid, and dash-dotted lines) and absence (blue line) of probiotics for three different probiotic growth rates. In the three cases shown, the pathogen growth rate is 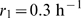, and the probiotic growth rate is 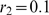, 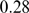, and 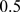 h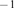, respectively. The initial number of probiotic bacteria in the lumen is 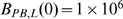 cells/g. Curves are shown for a small initial bacterial insult, 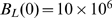 cells/g, for which the system converges to a health state for all parameter sets considered. This choice highlights the effects of 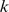 and 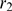 and the competition between probiotic and pathogenic bacteria in the lumen in the absence of bacterial translocation through the epithelium. In all cases, the curves generated with probiotics present intersect the line corresponding to the absence of probiotics at 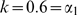 (the logistic growth competition parameter). Direct computation of 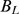 and 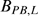 steady states from equations (1) and (2), with 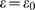, shows that if 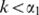, then probiotics are a beneficial treatment method since the steady state value of the sum 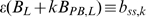 in the presence of probiotics is less than the steady state value of 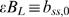 in the absence of probiotics. This outcome implies that 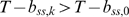. For 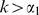, probiotics are harmful since 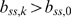. This outcome implies that threshold could be exceeded in the presence of probiotics even though this threshold is not exceeded in the absence of probiotics. For small 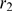 (dashed), the presence of probiotics has nearly no effect for 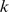 above some level, including 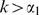, since pathogenic bacteria are predicted to outcompete probiotics in that parameter range. For sufficiently high 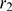 (dash-dotted), probiotics result in decreased luminal bacteria levels for 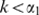 and elevated levels for 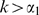.

**Figure 5:**
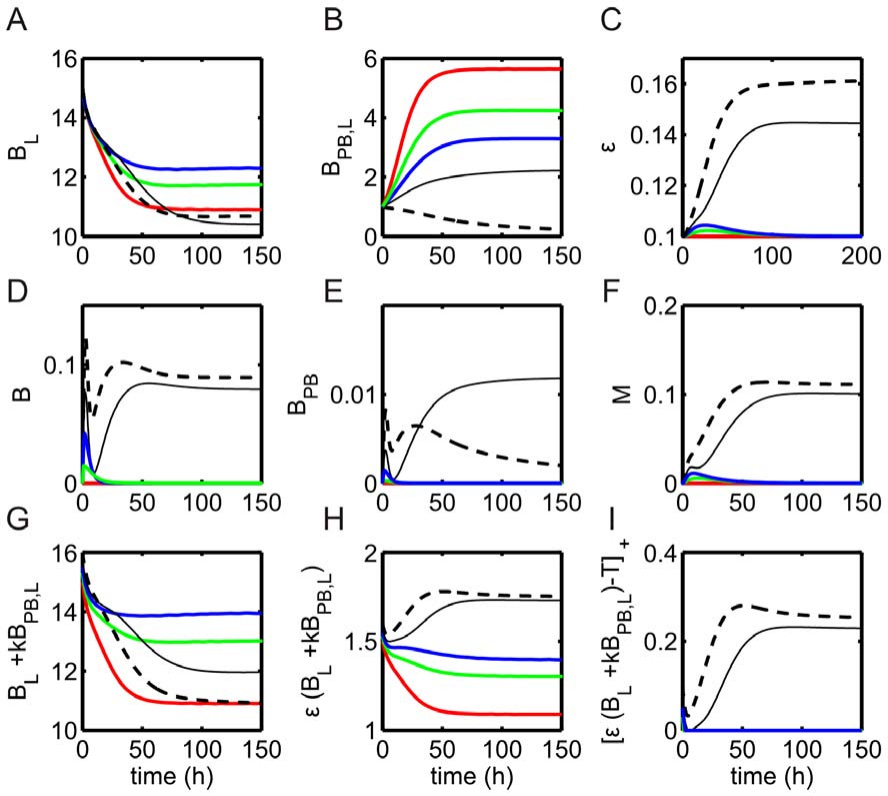
System dynamics in the presence of probiotics. Health or disease states are predicted as parameter 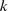 is varied: 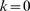 (red), 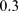 (green), 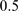 (blue), 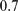 (black), and 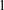 (dashed). The system is simulated in the bistable region, with initial pathogenic bacteria insult 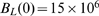 cells/g, pathogenic bacteria growth rate 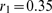 h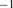, and probiotic bacteria growth rate 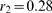 h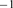. (A) Bacteria in lumen, 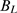. (B) Probiotic bacteria in lumen, 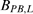. (C) Permeability, 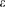. (D) Bacteria in blood/tissue, 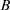. (E) Probiotic bacteria in blood/tissue, 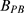. (F) Immune cells, 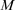. (G) Total bacteria in lumen, 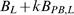. (H) Product of luminal bacteria and permeability, 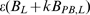. (I) Difference between product in (H) and threshold, 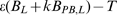.

Time dynamics for the system in the presence of probiotics are illustrated in Figure 5. The system is simulated in the bistable region, with 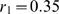 h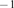, 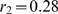 h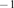, 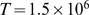 cells/mL/h, 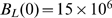 cells/g, and 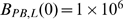 cells/g. Model predictions for multiple values of 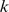 are shown: 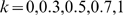. For 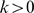, 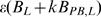 is above threshold at 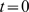 due to the initial levels of bacteria in the lumen, and thus there is an initial efflux of bacteria into the blood/tissue. For 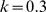 (green curve) and 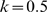 (blue curve), the inflammatory response is ultimately successful at eliminating bacteria in the blood/tissue, and the competitive effects of probiotics cause the overall number of bacteria in the lumen to be decreased from its initial value so that 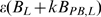 falls below threshold and bacterial permeability returns to the baseline value. Thus, the beneficial role of probiotic bacteria is evident as 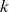 is decreased since probiotics are increased in the lumen, which causes pathogenic bacteria to be decreased in the lumen due to competition with probiotics and translocation into (and eventual elimination within) the blood/tissue compartment. For 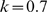 (black curve) and 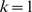 (dashed curve), however, 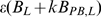 remains above threshold (panels H, I) and the observed decrease in luminal bacteria is due to the sustained efflux of bacteria into the blood/tissue. Interestingly, the steady state levels of both pathogenic and probiotic bacteria in the lumen are non-monotonic functions of 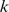, and increased permeability can maintain a disease state despite smaller luminal bacteria levels for large 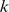. These results illustrate that model predictions of health and disease depend on the transient dynamics of bacteria in the lumen as well as the immune response and its consequences.

**Figure 6:**
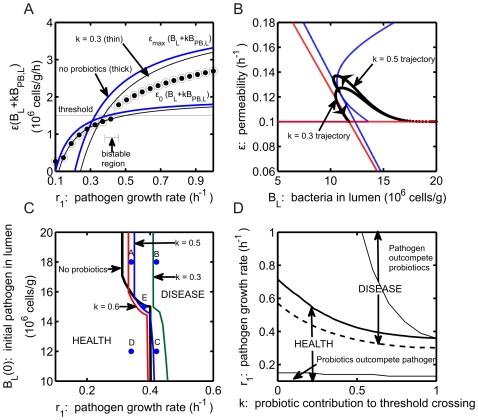
System behavior in the presence of probiotics. (A) Steady state values of bacteria and permeability in the presence of probiotics as the growth rate of pathogenic bacteria (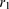) is varied. 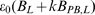 and 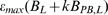 curves in the presence (black line, 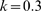) and absence (blue line) of probiotics are included. Steady state values of 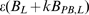, with 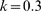, are given by (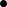) for 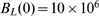 cells/g and () for 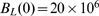 cells/g, as in Figure 3A. (B) 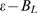 phase plane (magnified) corresponding to system dynamics in panel A with 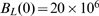 cells/g. The 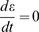 and 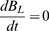 nullclines are shown for 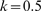 (blue) and 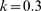 (red). Trajectories for 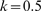 and 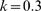 (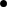, labeled) indicate predicted disease and health states, respectively. (C) Predictions of health and disease for various initial numbers of pathogenic bacteria (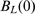) and pathogenic bacteria growth rates. Thick, black curve: separates regions of health and disease in the absence of probiotics. Solid curves separate regions of health and disease in the presence of probiotics with 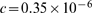 g/cell and 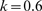 (red), 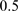 (blue), and 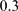 (green). System behavior is investigated at five points, A–E. (D) Predicted regions of health and disease are separated by a thick solid line and a dashed line, respectively, as parameters 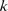 and 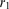 are varied. Bistability of stable health and disease states occurs for values of 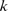 and 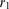 in the overlap of the health and disease regions. A summary of system dynamics is also included and separated by thin, solid curves.

The maximal and minimal steady state curves for the product of luminal bacteria and intestinal permeability in the absence of probiotics (Figure 6A: blue curves, as in Figure 3) are shifted to the right with respect to 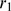 in the presence of probiotics, as illustrated in Figure 6A for 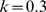 (black curves). As a result, the regions of disease and bistability occur at higher values of 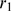, indicating the beneficial effect of probiotics on the system. This effect is also observed in Figure 6B, since the intersection point of the 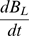 and 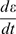 nullclines corresponding to a disease steady state is lost as parameter 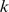 is decreased from 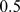 (blue) to 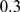 (red) (note that the initial condition 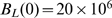 cells/g lies in the basin of attraction of the disease state for 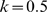).

Although the region of bistability shifts to larger 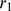 values with the introduction of probiotics, this rightward shift is less pronounced for larger 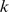. Moreover, the level of 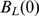 separating the basins of attraction of the health and disease states depends on 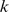 in addition to 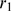. These trends can be seen in Figure 6C, which shows the boundary between initial conditions yielding health and those leading to disease as a function of 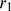 in the absence and presence of probiotics for various 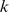 values and 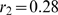 h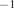. For any fixed 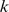, for low values of 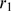, the inflammatory response successfully eliminates bacteria from the blood and tissue compartment so that a health state is always predicted. For high values of 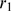, a level of bacteria persists in the blood/tissue, and a disease state is predicted. For intermediate values of 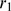, bistablity occurs, such that both health and disease outcomes are possible, depending on 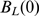. For bistable values of 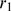, some 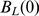 values that were in the health region without probiotics actually lie in the disease region with probiotics present, for sufficiently large 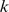 (e.g. 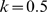 and 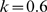 in Figure 6C). As 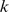 is decreased, probiotics contribute less to threshold crossing and the health region expands.

Five labeled points are included in Figure 6C to highlight the predicted model behavior for different bacterial initial conditions and virulence. At point A, which would have led to a disease state without probiotics, health is restored in the presence of probiotics with 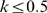. For a more virulent pathogen with the same 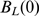, represented by point B, a disease state is always predicted by the model, irrespective of probiotic treatment (assuming 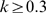). In general, in the absence of probiotics, an increase in the initial number of bacteria (from point D to A) or growth rate of pathogen (from point D to C) corresponds to a change from predicted health to predicted disease states. In the presence of probiotics with sufficiently small 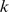, a health state is maintained despite traversing from points D to A or points D to C, demonstrating the benefit of probiotic treatment. However, point E lies in the region where the model predicts that probiotic treatment can actually be harmful, lowering the level of 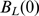 needed to induce disease, for a certain range of 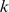. For this parameter set, probiotics contribute to threshold crossing in the model, enhancing the immune response and further increasing permeability in a way that is not resolved by subsequent decreases in luminal bacterial levels. The existence of such a region may help explain clinical studies in which probiotics did not reduce the incidence of NEC and in fact led to bacterial sepsis [21], [22].

Figure 6D provides a summary of predicted health and disease regions in the 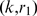 plane. The overlap in health and disease regions corresponds to the bistable region in which the initial degree of infection, 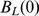, dictates the outcome. If 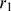 is small enough, then probiotics can outcompete pathogenic bacteria. However, for very virulent strains of pathogen (high 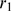 and 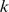), the pathogenic bacteria outcompete probiotics.

**Figure 7:**
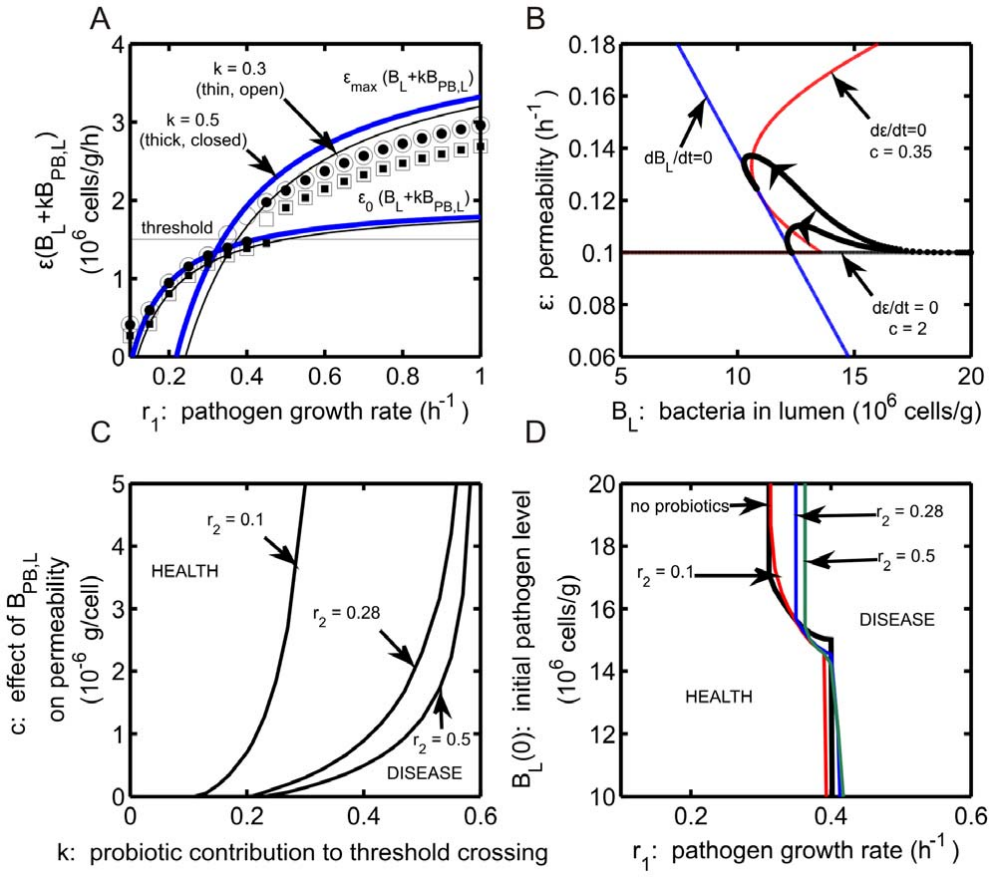
Effect of parameters 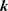 and 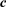 on system behavior. (A) System behavior for two 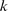 values (parameter relating the probiotic contribution to threshold crossing) as the growth rate of pathogenic bacteria (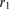) is varied. Curves as in Figures 3A and 5A. Steady state solutions of 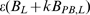 are shown for 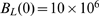 cells/g (closed symbols) and 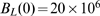 cells/g (open symbols) with 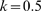 (circles) and 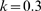 (squares). (B) 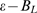 phase plane (magnified) as parameter 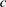 is varied in the system. The 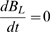 (blue) and 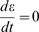 nullclines for 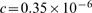 g/cell (red) and 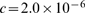 (black) are shown. Trajectories (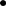) for both 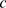 values are included. (C) Regions of health and disease predicted by the model as 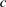 and 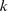 are varied. The system is initially in a disease state defined by 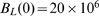 cells/g and 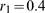 h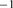. Combinations of 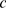 and 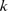 values above each curve represents regions in which health is restored. Values of parameter 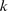 is varied in the range in which probiotics are predicted to be beneficial: 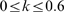. Curves for different probiotic bacteria growth rates (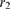) are included: 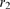 = 0.1, 0.28, and 0.5 h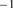. (D) Effect of initial number of pathogenic bacteria (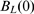) and probiotic bacteria growth rate (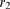) on predictions of health and disease is shown as 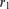 is varied. Thick black curve: separates regions of health and disease in the absence of probiotics. The following curves separate regions of health and disease in the presence of probiotics with 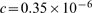 g/cell and 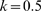: 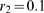 h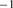 (red), 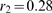 h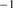 (blue), and 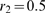 (green).

Based on our model formulation, as 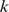 is decreased, probiotics become progressively more beneficial to the system. In addition, as 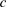 is increased, epithelial permeability to bacterial translocation is reduced, which also promotes health. These effects are consistent with the natural expectation that probiotic strains characterized by a small 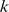 value (corresponding to a low tendency toward epithelial translocation) and a large 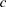 value (representing strong anti-inflammatory effects on epithelial permeability) are likely to yield the optimal treatment outcome. Just as seen with the introduction of probiotics in Figure 6A, a decrease in 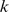 shifts the steady state bounds on luminal bacteria levels to the right with respect to 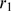. Curves for 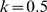 (blue curves, circles) and 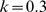 (black curves, squares) are shown in Figure 7A. Disease and bistability are predicted to occur at higher values of 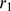 for decreased 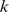 values, yielding a larger region of predicted health. Changes in 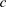 affect the location of the 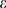-nullcline. In Figure 7B, a shift in the 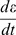 nullcline is evident for increased 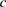 values. The red curve indicates the 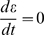 nullcline for 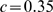; if 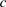 is increased to 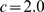, the 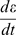 nullcline (black) lies entirely at 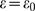 and only healthy outcomes can result for all 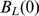 in the range shown. The tradeoff of parameters 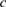 and 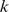 is investigated in Figure 7C for an initial state that would yield disease in the absence of probiotics (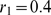 h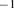 and 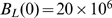 cells/g). The regions of predicted health and disease are identified for three different values of probiotic growth rate, 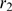. Regions above the given curve correspond to combinations of parameters 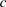 and 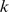 that yield predictions of health, and regions below each curve correspond to disease. As 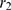 increases, a greater region of health is predicted since more probiotics are present in the system. A health state independent of the value of 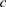 is predicted for 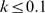, given the parameter values considered here. We observe greater sensitivity to 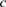 when 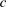 is small than when it is large, suggesting that effects of probiotics on epithelial permeability are saturating, while sensitivity to 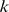 dominates once 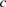 is large enough.

The effect of 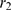 on the curves separating health and disease in the 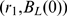 plane with 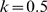 is shown in Figure 7D. Due to the threshold term in equation (5), increasing the growth rate of probiotics is expected to have a similar effect as increasing the effectiveness of probiotics (i.e., decreasing parameter 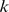), and indeed Figure 7D is very similar to Figure 6C. In general, as 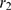 is increased from 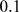 to 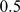 h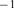, the region of predicted health increases. However, as is evident from the intersection of the curves in the bistable region, at least for small 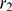, there are some values of 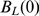 for which health would have resulted in the absence of probiotics yet disease is predicted with probiotics.

**Figure 8:**
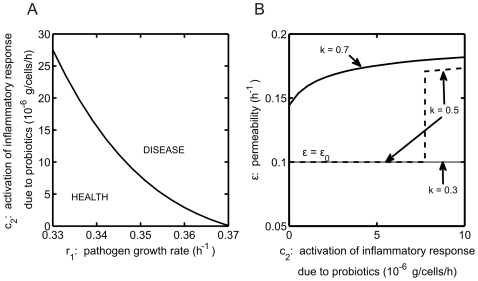
Interplay of probiotics and inflammatory response. (A) Model predictions of health and disease as parameters 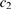 (the activation of the inflammatory response due to the presence of probiotic bacteria in the blood/tissue) and 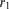 (the growth rate of pathogenic bacteria) are varied. System is simulated in the bistable region, with initial pathogenic bacteria insult 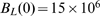 cells/g, probiotic contribution to threshold crossing 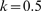, and probiotic bacteria growth rate 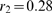 h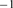. (B) Effect of inflammatory response activation by probiotic bacteria (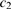) on the permeability of the intestinal wall (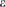). Baseline permeability is 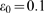 h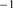. Parameter 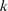 is varied: 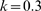, 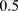, and 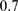 (labeled).

The interplay between probiotics and the activation of the inflammatory response is investigated in Figure 8. While an increased inflammatory response helps the system to defeat an invading pathogen, the inflammation that accompanies the inflammatory response causes damage to the intestinal barrier, thereby increasing the permeability 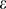 of the layer. Parameter 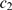 gives a measure of immune activation due to probiotics. System behavior for various 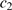 values is illustrated in Figure 8. The system is assumed to be initially in the bistable region, 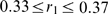 h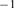, with 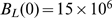 cells/g. In panel A, for each fixed 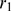, a disease outcome is predicted once parameter 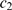 exceeds a certain level, which decreases as 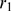 increases. For 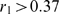 h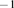, disease is predicted for all values of 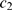, and when 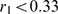 h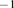, health is predicted unless 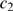 is increased outside of the biologically relevant regime. In panel B, the system is also simulated in the bistable region with 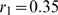 h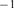. We chose 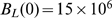 cells/g, which yields health for all 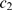 for 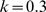. As 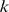 increases, the outcome depends on 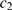. Health is lost at a fixed value of 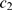 for 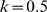, because additional immune activation leads to too much intestinal permeability to overcome. If 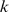 is sufficiently large, such as 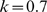, then disease results for all 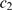, with the steady-state value of 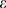 increasing as a function of 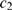.

### Probiotic dosing

**Figure 9:**
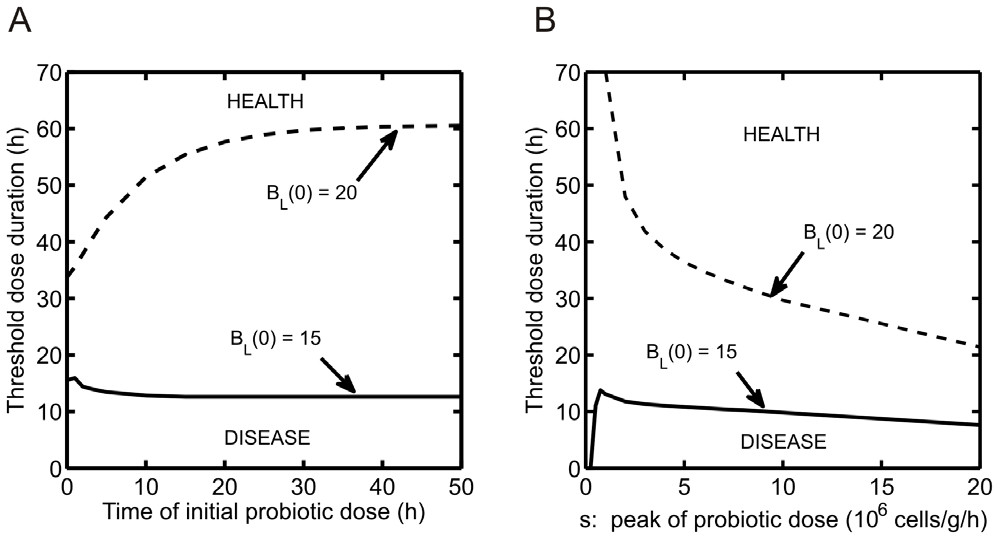
Effect of peak, duration, and timing of administration of probiotics. Curves denote minimal duration for which a dose of probiotics (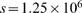 cells/g/h) must be adminstered to result in health (defined as threshold dose duration). Two different initial bacteria levels are considered: 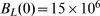 cells/g (solid) and 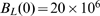 cells/g (dashed). In all simulations, 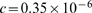 g/cells and 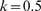. (A) Change in the threshold dose duration for probiotic administration as the time of administration is varied. (B) Change in the threshold dose duration for probiotic administration as dose level (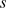) is increased.

Our model predicts that the time, duration, and dose level of probiotic administration can determine its effectiveness at restoring health. A probiotic dose is simulated in the model by adding a constant 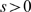 to the right hand side of equation (2) for a fixed time period. In Figure 9, the minimal duration of probiotic dose required to yield a health state is investigated as the initial time of probiotic administration and the probiotic dose levels are varied. These effects are studied for two different initial levels of pathogenic bacteria. Points above the curves correspond to dosing parameters yielding a health state. In Figure 9A, the dashed curve corresponds to an initial disease state given by the following conditions and parameters: 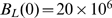 cells/g, 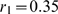 h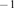, 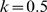, and 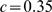. A probiotic dose of 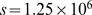 cells/g/h is used in panel A. The length of time for which probiotics must be administered at a particular dose in order to restore health (defined here as the threshold dose duration) is predicted to increase as the time at which probiotics are administered is delayed. This outcome is expected, since administering probiotics for a shorter period of time will be effective in a system that has not yet reached a steady state value for disease. Once a steady state is reached, the necessary threshold dose duration does not change. A negative relationship between threshold dose duration and time of administration is predicted when 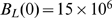 cells/g, 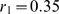 h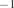, 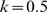, and 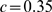 (solid curve). In this case (Figure 6C, point E), probiotics can have the harmful effect of lowering the level of 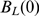 needed for disease to result. Waiting before giving probiotics allows 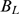 to decrease on its own, such that the threshold dose duration decreases. In Figure 9B, results are shown from simulations with the same initial conditions as in panel A. The threshold dose duration required to restore health is predicted to decrease as the probiotic dose level is increased. However, if the system is initially in the part of the bistable region in which the presence of probiotics is harmful to the system (as in Figure 6C, point E), then the threshold dose duration required to restore health first increases with dose level before it decreases.

## Discussion

The model presented in this study represents a preliminary tool for exploring the effects of probiotic treatment for NEC. The model incorporates several experimentally supported mechanisms through which probiotics can mitigate effects of pathogenic bacteria on the immature gut. Specifically, probiotics affect the number of pathogenic bacteria in the lumen as well as the overall number of bacteria there, the degree of epithelial wall permeability, the number of bacteria in the blood/tissue, and the activation of the inflammatory response. We simulated the model equations for different levels of initial pathogenic insult, 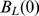, and different parameter values associated with the relative strengths of these mechanisms.

### Dependence of model dynamics on parameters associated with probiotics

Mattar et al. [33] showed that the presence of probiotics in the intestinal lumen generally leads to a decrease in the level of pathogen in the lumen. Our results agree with this finding, assuming 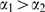. Figure 6D shows that probiotics can outcompete pathogenic bacteria if the growth rate of these pathogens is small, while a high enough pathogen growth rate can allow these bacteria to predominate over probiotics. We find that under most conditions, the two species coexist in the lumen. For a fixed threshold parameter 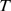, the translocation of bacteria is determined by the product of two factors, namely the effective size of the luminal bacteria population 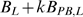 and the epithelial permeability 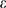. As seen in Figure 4, as long as the parameter 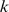 is below 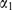, the presence of probiotics decreases the steady state value of this product. This decrease may allow the system to avoid bacterial efflux into the blood/tissue and the associated inflammatory response, or it may allow the system to exhibit a weaker flux of bacteria through the epithelium if a lowering of the epithelial permeability threshold were to occur. The ability of probiotics to decrease epithelial permeability itself, which is analogous to our 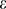, was verified by Kennedy et al. [10] and is demonstrated in Figure 7, in which a health state is promoted as parameter 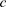 (a measure of the probiotic effect on permeability) is increased. As a result of these effects, the number of bacteria in the blood/tissue is predicted to be decreased. However, even if pathogenic and probiotic bacteria enter the blood/tissue compartment, probiotics activate inflammation to a lesser degree than do pathogenic bacteria. This effect is observed experimentally [32] and in our model is due to the assumption that 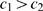 in equation (6).

The most interesting feature of our model's dynamics is the bistability between health and disease states that occurs over a range of pathogenic growth rates in the transition between health-only and disease-only regimes. The epithelial barrier is a key component of this bistability. Specifically, this barrier prevents activation of the inflammatory response when the number of luminal bacteria is below a threshold [30], allowing for a stable health state. For the same parameter values, however, a transient elevation in the number of pathogenic bacteria that leads to translocation across the epithelial barrier stimulates an inflammatory response. This response can be advantageous, since the inflammatory cells eliminate pathogenic bacteria, yet the activation of these inflammatory cells also enhances epithelial permeability and effectively reduces the threshold. Combined, these two processes can result in the emergence of a stable disease state (Figure 8). The introduction of probiotics into the lumen yields a decrease in the total size of the steady state bacterial population in the lumen in the absence of threshold crossing (Figure 4 and 5G). Probiotics may contribute to a transient elevation in total luminal bacteria, however, which may produce an efflux into the blood/tissue when this threshold crossing effect is taken into account. Thus, the presence of probiotics may negatively impact patients by paradoxically lowering the level of pathogenic bacteria required to induce a disease outcome. The larger the value of parameter 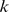, the lower this necessary number of pathogenic bacteria becomes (Figure 6C). Clinical studies have shown both positive and negative outcomes when probiotics are administered to premature infants [18]–[22]. Points A–E in Figure 6C have been selected to illustrate how outcomes of different treatment strategies could depend quite sensitively on the size of an initial pathogenic insult, the virulence of the pathogen, and the characteristic 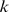 of the probiotics. At points A, C, and E, both health and disease states are possible and depend on initial conditions and parameters, while at B and D, outcome is independent of probiotics for the parameter range considered. At point E, probiotic administration converts a health outcome to a disease outcome, while the opposite can be true at A and C, depending on the nature of the probiotics applied. This sensitivity suggests that multiple conditions should be tested clinically in efforts to identify the potential benefit and harm of probiotic treatment.

### Modeling specific patient populations and interventions

In addition to infection type and severity, many experiments have indicated that the effectiveness of probiotic treatment on the incidence of NEC may also depend on feeding type, delivery type, and health disorders of the infant (e.g., hypoxia). For example, studies have shown that breast-fed infants acquire a more desirable intestinal flora than formula-fed infants, since breast milk contains many antimicrobial products and factors that promote the colonization of helpful bacteria in the infant intestine [3], [34], [35]. In fact, a 10-fold increase in the incidence of NEC was found in formula-fed infants compared with breast-fed infants [4]. The effects of breast-feeding could be simulated using our mathematical model by decreasing the growth rate of pathogenic bacteria (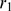), decreasing the damage caused by the inflammatory response (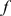), decreasing the carrying capacity of pathogenic bacteria (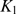), and decreasing the baseline epithelial permeability (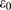). It is important to note, however, that, since breastfeeding is the biological norm for the infant digestive system, these adjustments should be thought of as restoring the model system to a baseline state, whereas the parameters used throughout this paper represent a perturbation to this baseline state, associated with the regime in which NEC is likely to occur. Clearly, different interventions should be designed for formula-fed versus breast-fed infants, given the differences between these two populations.

Studies have also shown that infants born vaginally tend to be colonized earlier with beneficial species of bacteria, while infants delivered by cesarean section have a delayed colonization by desirable bacteria [1], [4]. In our model, the initial population of luminal probiotic bacteria could be assumed to be higher in infants born vaginally to distinguish birth type. In addition, premature infants with NEC are often treated with antibiotics and other methods that aim to reduce their load of pathogenic bacteria. These interventions, however, also affect their normal gut colonization and can increase NEC severity. The effects of antibiotics have been included in previous models of infection and acute inflammation [25] and can be simulated in the current model by decreasing parameters 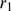 and 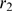, the growth rates of pathogenic and probiotic bacteria. Importantly, the killing of bacteria with antibiotics may release factors that trigger the release of immunostimulants, thereby contributing to the overwhelming degree of inflammation observed in NEC. NEC has been observed occasionally in full-term babies but is often associated with infants suffering from cyanotic congenital heart disease, a hypoxic-ischemic event, polycythemia, or *in utero* growth restriction [3], [4]. These diseases are associated with a history of hypoxia, in which the resulting decrease in blood supply may affect the integrity of the intestinal lining. To account for a hypoxic event in the model, the baseline level of epithelial permeability 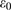 could be increased. Because our model has been developed such that key parameters control effects of infection and of probiotic treatment, it can be used to investigate various experimental observations through adjustments in parameter values and initial conditions. Although the model has been developed specifically to address the incidence of NEC in neonates, many gastrointestinal diseases exhibit similar mechanisms and characteristics, and thus this model may also be adapted to investigate other gastrointestinal disorders in a variety of age groups.

Determining the correct probiotic dosing strategy is a key question for the realization of effective probiotic treatment for infants suffering from NEC. Our mathematical model predicts that probiotics will be most effective for low rates of pathogenic growth (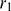), moderate rates of probiotic growth (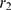), high levels of probiotic reduction of epithelial permeability (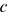), and a low ability of probiotics to cross the epithelial barrier (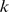). In clinical studies of probiotic supplements administered to pre-term neonates, the time at which probiotics are administered varies between 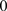 and 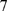 days of birth [3], [18], [20], [21]. Also, the studies implement different numbers of doses per day and include multiple probiotic species. It is hypothesized that treatment with a mixture of probiotic strains as opposed to a single strain may have an improved effect on preventing NEC in premature infants [16]. In future work, information obtained from simulating the model using different dosing regimens (Figure 9) and different initial conditions and parameter values (Figure 6C), customized to represent particular probiotic treatment conditions, may be used to predict outcomes of probiotic treatment strategies. Moreover, an optimal control approach may be applied to the model to generate optimal dosing time courses.

### Additional considerations and conclusions

Our main motivation in designing this study was to incorporate experimental observations of probiotics into a mathematical model that can be used to gain insight into key interactions of pathogens, probiotics, and the inflammatory response in the context of NEC. In this way, we hope to improve clinical translation, as part of our larger Translational Systems Biology framework [29], [36]–[39]. In particular, we have included mathematical terms in our model that represent important effects that have been implicated in the development of NEC and some of the mechanisms through which probiotics are thought to act to effect its progression [1], [14]. We have utilized this model to suggest specific reasons why probiotics might be harmful, for example by paradoxically lowering the level of pathogenic bacteria required to induce a disease outcome, and to highlight the features that characterize beneficial probiotics.

Our basic modeling assumption is that the inflammatory response that takes place at the lumen/blood interface, and that involves an interplay among intestinal flora, intestinal epithelial cells, and inflammatory cells in the blood, serves to maintain a dynamic equilibrium that defines the health steady state. It is likely that an effective inflammatory response requires some small, baseline rate of efflux of luminal bacteria into the blood/tissue. The ensuing minor, self-limiting inflammatory response may serve to maintain the mostly beneficial population of intestinal bacteria while providing a sampling of intestinal contents that could lead to an early warning of changes in the proportion of pathogenic bacteria in the intestinal lumen. F or a developing infant, this equilibrium may require a constant influx of factors present in maternal breast milk. To incorporate such a baseline inflammatory response, which we currently omit, the model should be augmented to include the roles of pro- and anti-inflammatory cytokines in the inflammatory response. One important effect of anti-inflammatory cytokines is the reduction of damage to the epithelium caused by the inflammatory response. In our current model, the omission of anti-inflammatory cytokines provides a worst-case scenario with respect to the harmful effects of the inflammatory response. The qualitative relationships established in this study that indicate both beneficial and harmful effects of probiotics are still expected to hold in the presence of cytokines, but additional insight into the interplay of the immune response and probiotic treatment will require future modeling of cytokine populations [40], [41].

The number of experimental and clinical studies that have been performed for NEC is limited due to the nature of the disease and the complexity of carrying out studies and obtaining samples in pre-term infants, and thus we used a combination of human and animal studies to provide an experimental grounding for the model presented. Additional experimental data would help to determine some of the parameter values estimated in this study and may also highlight additional factors contributing to NEC that have not been explored by this model. Interactions between bacteria and the inflammatory response are defined within the context of two lumped compartments that are assumed to be well-mixed, and thus there is no spatial component in this present model. Immune mechanisms specific to regions such as the gut mucosa and lamina propria [31] are not included explicitly. A more mechanistic representation of the threshold for epithelial permeability would also improve our model, although further experiments are needed to provide relevant details. Interestingly, a recent simulation study does suggest that the intensity of the inflammatory response does depend on the phenomenon of pathogenic growth [42], in line with our threshold-based dependence of inflammatory activation on the extent of pathogenic proliferation. Indeed, Hooper and Macpherson [31] suggest that if the luminal bacterial load remains below a certain “numerical threshold,” then an inflammatory response is not evoked. Overall, the hypotheses formulated using this model must be tested with experimental work to establish under what conditions, and through what mechanisms, probiotics can yield beneficial effects as a treatment for NEC.

In conclusion, based on experimental and clinical studies, we have developed a simplified mathematical model of the complex host-pathogen interaction that occurs in the setting of NEC and used it to analyze the impact of probiotic administration on the ensuing dynamics. The predictions derived from this computational study may help to explain the diverse outcomes that may arise in this setting and may be useful for guiding future experimental and clinical studies.

## References

[1] Claud EC, Walker WA (2008). Bacterial colonization, probiotics, and necrotizing enterocolitis.. J Clin Gastroenterol.

[2] Guner YS, Friedlich P, Wee CP, Dorey F, Camerini V (2009). State-based anaylysis of necrotizing enterocolitis outcomes.. J Surg Res.

[3] Lin PW, Nasr TR, Stoll BJ (2008). Necrotizing enterocolitis: Recent scientific advances in pathophysiology and prevention.. Semin Perinatol.

[4] Hunter CJ, Upperman JS, Ford HR, Camerini V (2008). Understanding the susceptibility of the premature infant to necrotizing enterocolitis.. Pediatr Res.

[5] Kosloske AM, Burstein J, Bartow SA (1980). Intestinal obstruction due to colonic stricture following neonatal necrotizing enterocolitis.. Ann Surg.

[6] Ricketts RR, Jerles ML (1990). Neonatal necrotizing enterocolitis: experience with 100 consecutive surgical patients.. World J Surg.

[7] Koffeman GI, vanGemert WG, George EK, Veenendaal RA (2003). Classification, epidemiology and aetiology.. Best Pract Res Clin Gastroenterol.

[8] Petty JK, Ziegler MM (2005). Operative strategies for necrotizing enterocolitis: the prevention and treatment of short-bowel syndrome.. Semin Pedriatr Surg.

[9] Reynolds A, Rubin J, Clermont G, Day J, Vodovotz Y (2006). A reduced mathematical model of the acute inflammatory response. I. Derivation of model and analysis of anti-inflammation.. J Theo Bio.

[10] Kennedy RJ, Kirk SJ, Gardiner KR (2002). Mucosal barrier function and the commensal flora.. Gut.

[11] Garcia-Lafuente A, Antolin M, Guarner F, Crespo E, Malagelada JR (2001). Modulation of colonic barrier function by the composition of the commensal flora in the rat.. Gut.

[12] Hammerman C, Kaplan M (2006). Probiotics and neonatal intestinal infection.. Curr Opin Infect Dis.

[13] Gribar SC, Sodhi CP, Richardson WM, Anand RJ, Gittes GK (2009). Reciprocal expression and signaling of TLR4 and TLR9 in the pathogenesis and treatment of necrotizing enterocolitis.. J Immunology.

[14] Barclay AR, Stenson B, Simpson JH, Weaver LT, Wilson DC (2007). Probiotics for necrotizing enterocolitis: A systematic review.. JPGN.

[15] Millar M, Wilks M, Costeloe K (2003). Probiotics for preterm infants?. Arch Dis Child Fetal Neonatal Ed.

[16] Szajewska H, Setty M, Mrukowicz J, Guandalini S (2006). Probiotics in gastrointestinal diseases in children: hard and no-so-hard evidence of efficacy.. JPGN.

[17] AlFaleh K, Bassler D (2008). Probiotics for prevention of necrotizing enterocolitis in preterm infants..

[18] Hoyos AB (1999). Reduced incidence of necrotizing enterocolitis associated with enteral administration of *Lactobacillus acidophilus* and *Bifidobacterium infantis* to neonates in an intensive care unit.. Int J Infect Dis.

[19] Lin H, Hsu C, Chen H, Chung M, Hsu J (2008). Oral probiotics prevent necrotizing enterocolitis in very low birth weight preterm infants: A multicenter, randomized controlled trial.. Pediatrics.

[20] Bin-Nun A, Bromiker R, Wilschanski M, Wilschanski M, Kaplan M (2005). Oral probiotics prevent necrotizing enterocolitis in very low birth weight neonates.. J Pediatr.

[21] Dani C, Biadaioli R, Bertini G, Martelli E, Rubaltelli FF (2002). Probiotics feeding in prevention of urinary tract infection, bacterial sepsis and necrotizing enterocolitis in preterm infants. A prospective double-blind study.. Biol Neonate.

[22] Land MH, Rouster-Stevens K, Woods CR, Cannon ML, Cnota J (2005). *Lactobacillus* sepsis associated with probiotic therapy.. Pediatrics.

[23] Day J, Rubin J, Vodovotz Y, Chow CC, Reynolds A (2006). A reduced mathematical model of the acute inflammatory response. II. Capturing scenarios of repeated endotoxin administration.. J Theo Bio.

[24] Kumar R, Clermont G, Vodovotz Y, Chow CC (2004). The dynamics of acute inflammation.. J Theo Bio.

[25] Kumar R, Chow CC, Bartels JD, Clermont G, Vodovotz Y (2008). A mathematical simulation of the inflammatory response to anthrax infection.. Shock.

[26] Chow CC, Clermont G, Kumar R, Lagoa C, Tawadrous Z (2005). The acute inflammatory response in diverse shock states.. Shock.

[27] Hunter CJ, Williams M, Petrosyan M, Guner Y, Mittal R (2009). *Lactobacillus bulgaricus* prevents intestinal epithelial cell injury caused by *Enterobacter sakazakii*-induced nitric oxide both *in vitro* and in the newborn rat model of necrotizing enterocolitis.. Infect and Immun.

[28] Matzinger P (2002). The danger model: a renewed sense of self.. Science.

[29] Vodovotz Y, Constantine G, Rubin J, Csete M, Voit E (2009). Mechanistic simulations of inflammation: Current state and future prospects.. Math Biosci.

[30] Han X, Fink MP, Yang R, Delude RL (2004). Increased iNOS activity is essential for intestinal tight junction dysfunction in endotoxemic mice.. Shock.

[31] Hooper LV, Macpherson AJ (2010). Immune adaptations that maintain homeostasis with the intestinal microbiota.. Nature Reviews Immunology.

[32] Hooper LV, Gordon JI (2001). Commensal host-bacterial relationships in the gut.. Science.

[33] Mattar AF, Drongowski RA, Coran AG, Harmon CM (2001). Effect of probiotics on enterocyte bacterial translocation in vitro.. Pediatr Surg Int.

[34] Schanler RJ (2001). The use of human milk for premature infants.. Pedr Clin NA.

[35] Lucas A, Cole TJ (1990). Breast milk and neonatal necrotising enterocolitis.. Lancet.

[36] An G, Faeder J, Vodovotz Y (2008). Translational systems biology: Introduction of an engineering approach to the pathophysiology of the burn patient.. J Burn Care Res.

[37] Vodovotz Y, Csete M, Bartels J, Chang S, An G (2008). Translational systems biology of inflammation.. PLoS Comput Biol.

[38] An G, Mi Q, Dutta-Moscato J, Solovyev A, Vodovotz Y (2009). Agent-based models in translational systems biology.. WIRES.

[39] Vodovotz Y, Constantine G, Faeder J, Mi Q, Rubin J (2009). Translational systems approaches to the biology of inflammation and healing.. Immunopharmacol.Immunotoxicol.

[40] Edelson MB, Bagwell CE, Rozycki HJ (1999). Circulating pro- and counterinflammatory cytokine levels and severity in necrotizing enterocolitis.. Pediatrics.

[41] Markel TA, Crisostomo PR, Wairiuko GM, Pitcher J, Tsai BM (2006). Cytokines in necrotizing enterocolitis.. Shock.

[42] Bewick S, Yang R, Zhang M (2009). The danger is growing! A new paradigm for immune system activation and peripheral tolerance.. PLoS ONE.

